# Supplementary figures and images for: Machine learning for the prediction of urosepsis using electronic health record data
Source: PLOS Digit Health. 2025 Jul 3;4(7):e0000896. doi: 10.1371/journal.pdig.0000896 (PMC12225808; doi:10.1371/journal.pdig.0000896)

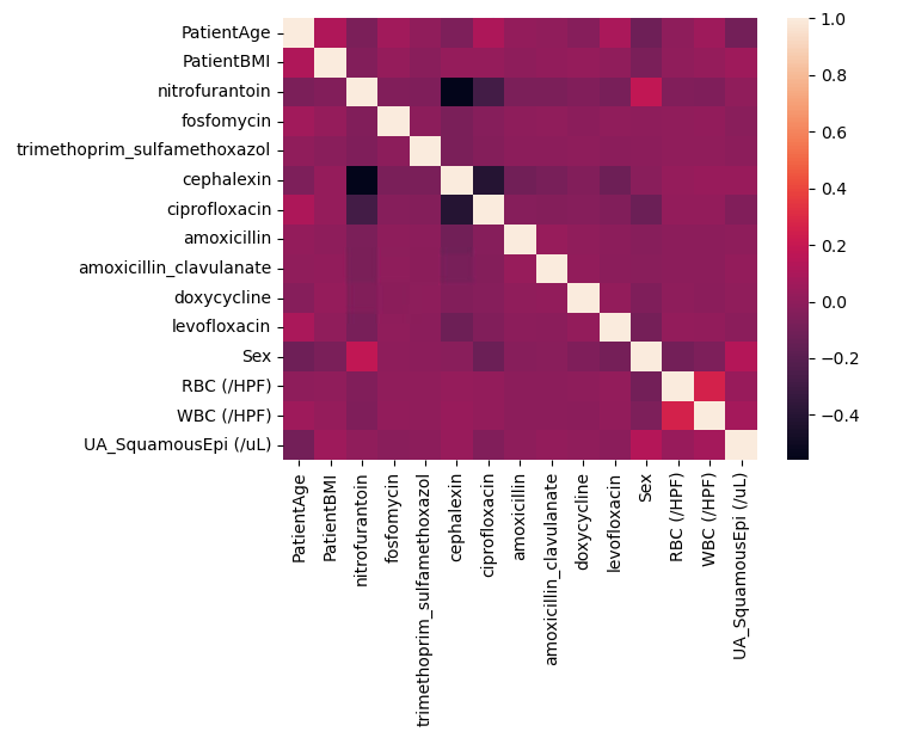

Supplement: S1 Fig — Heatmap showing pairwise Pearson correlations between model features. Color intensity represents correlation strength, with darker colors indicating stronger correlations. (TIFF) [file pdig.0000896.s003.tiff]
