# Supplementary material for: Machine learning for the prediction of urosepsis using electronic health record data
Source: PLOS Digit Health. 2025 Jul 3;4(7):e0000896. doi: 10.1371/journal.pdig.0000896 (PMC12225808; doi:10.1371/journal.pdig.0000896)
Supplement: Attachment — Submitted filename: PLOS_Digital_Health_Review_PDIG-D-24-00227.pdf [file pdig.0000896.s007.pdf]

PLOS Digital Health Review: Machine learning for the prediction of urosepsis using electronic health record data

Manuscript Number: PDIG-D-24-00227.

The authors proposed machine learning models to predict hospital admission and urosepsis for patients with an outpatient UTI encounter.

1 Abstract

1. The abstract is too lengthy and should be summarized to include only the main points. Please ensure that the manuscript meets with the submission guidelines.
2. "Our models demonstrated high predictive performance with an area under the ROC curve (AUC) of 79.5% AUC and an area under the precision-recall curve (APR) of 13% APR for reencounters" - PR AUC of 13% sounds not high and ROC is less relevant for imbalance binary classification as mentioned in the Method section. Can you explain the claim that these results are high predictive performance? In addition, please clarify what are the baselines that the results with high predictive performance are compared to.
3. Consider to add to the Abstract the names of the Machine Learning models that were used for the predictions.

2 Introduction

1. There is a previous work by Zhang et al. (2021) [1] that appears highly relevant to this research but is not mentioned in the related work section of the introduction.

3 Methods

1. Please improve the resolution of the figures, such as Figure 1 from this section, as well as Figures 2, 3, and 4.
2. Methods, third paragraph: "We split our examination into two major analyses. First, we predicted the risk of a patient with a UTI being admitted to the hospital for any reason in a 7-day window. Next, we narrowed down our predictor variable to consider only reencounters that were demonstrated cases of urosepsis" - Adding an ablation study with a baseline that predicts directly without the first step ("First, we predicted the risk of a patient with a UTI being admitted to the hospital for any reason"), can be beneficial for analysis and comparison.
3. In addition to the previous comment, please provide a clarification on the hypothesis behind splitting this examination into two steps.
4. The train and test split is in a 75:25 ratio, and the validation set was later used for hyperparameter tuning. Please clarify the source of the validation set.
5. Consider adding general statistics that provide an overview of the task and dataset. For example, include the percentage of UTI patients diagnosed with urosepsis and those who are not. This will give a better estimate of how imbalanced the classification task is. The

analysis of the Results (Data and data processing subsection) is more relevant to the Method section.

6. Please justify the choice of these three machine learning models: logistic regression, decision tree, and random forest. For example, explain why XGBoost [2] was not used.
7. The data is very imbalanced. Adding an approach to augment the dataset with more positive examples can enhance the contribution of this work.
8. Have the authors considered any approaches to address the data imbalance? in a scenario of significant data imbalance, exploring approaches that address imbalanced datasets is highly relevant and essential to include in the manuscript.

4 Results

1. There are some repetitions in the paper. For example, the train-test split ratio is mentioned both in the Methods and Results sections.
2. Adding a caption to Figure 2 can enhance readability.
3. Missing baselines: consider adding a random classifier and an always positive or negative classifier as baselines for comparison.
4. The reproducibility of this work is limited since the dataset is private. Therefore, consider applying your method to other datasets as well, especially public datasets under basic restrictions such as MIMIC-IV or eICU-CRD if possible. Additionally, reporting the results of another dataset can enhance the generalizability of this work.
5. Consider to include the confidence intervals as part of the results.

5 Discussion

1. Paragraph two: "but demonstrated that random forests were the best performing models, both in terms of ROC and model interpretability" - In a task where the data is highly imbalanced, ROC indeed is not relevant. Moreover, I think ROC performance can be misleading for that task given the extreme data imbalance. In light of this, I think it is better to remove this metric from the results. APR is more relevant, and reporting the precision and recall of the positive label (at a specific threshold) might also be interesting for real-world scenarios.
2. It is worth adding to the discussion whether, in your opinion, a practical application of predicting this task using this model is possible, considering the results, and how it compares to the current situation.
3. Error analysis can be beneficial to understand the errors of the models. For example, it can be interesting to see what errors the Random Forest made in Figure 2B.
4. A more comprehensive discussion is needed to thoroughly analyze the result graphs. For example, how do you explain the very low accuracy in logistic regression, and what is the practical effect of the metrics, particularly in Figure 2B? Can a Random Forest model that achieves low recall and high precision be beneficial?
5. In addition, the analysis lacks references to other baselines that were not tested in this paper, such as a model that always gives the same answer or a random classifier.
6. It is worth comparing the results achieved by humans in this task with those presented in the manuscript.

References

[1] Luming Zhang, Feng Zhang, Fengshuo Xu, Zichen Wang, Yinlong Ren, Didi Han, Jun Lyu, and Haiyan Yin. “Construction and evaluation of a sepsis risk prediction model for urinary tract infection”. In: *Frontiers in Medicine* 8 (2021), p. 671184.

[2] Tianqi Chen and Carlos Guestrin. “Xgboost: A scalable tree boosting system”. In: *Proceedings of the 22nd acm sigkdd international conference on knowledge discovery and data mining*. 2016, pp. 785–794.
