# Supplementary material for: Machine learning for the prediction of urosepsis using electronic health record data
Source: PLOS Digit Health. 2025 Jul 3;4(7):e0000896. doi: 10.1371/journal.pdig.0000896 (PMC12225808; doi:10.1371/journal.pdig.0000896)
Supplement: Attachment — Submitted filename: Urosepsis ResponsetoReviewers.docx [file pdig.0000896.s009.docx]

Responses to Reviewers

PLOS Digital Health: Machine learning for the prediction of urosepsis using electronic health record data

***Reviewer #1:*** *The study developed a machine learning model for predicting the risk of hospitalization and urosepsis in outpatient urinary tract infection patients. Standardized disease coding with ICD-10 was utilized for the included conditions, and the model achieved satisfactory predictive performance. However, there are several limitations in this research:*

1. *In 2016, sepsis was defined by new diagnostic criteria, and the R65 code used in the study stands for systemic inflammatory response syndrome. The urosepsis cases discussed in the study actually encompass both patients with positive blood culture for sepsis and those with systemic inflammatory response syndrome, indicating the presence of confounding factors among the urosepsis patients in this study.*

We thank the reviewer for bringing this up. The following explains why we defined urosepsis as both positive blood culture, and inflammatory response. When patients present with bacteriuria, the consensus for determining who should be treated with antibiotics for potential urinary tract infection centers on whether the patient has “signs or symptoms attributable to the urinary tract”. The exception to this rule is if patients are exhibiting any signs that could be consistent with systemic infection or bacteremia, in whom it is the standard of care to treat a positive urine culture. There is no specific ICD code for urosepsis specifically, so we intentionally chose broad definitions for those patients who would meet the criteria for any suspicion of sepsis/bacteremia, which we felt would reasonably include this population and account for any possible miscoding of an individual at presentation. As most of the diagnostic codes were made at the time of the initial presentation, we felt it possible that these codes could reflect the situation in which a patient was later found to have sepsis. If they never had a positive blood culture, they would not have been included in the population, so we felt it was reasonable to try and account for possible miscoding at diagnosis. Throughout the literature, this combination of codes, including R65, has been used to ensure the capture of populations with systemic infections, especially given non-specialist lack of familiarity with proper coding at diagnosis when only incomplete information is available (PMIDs: 27839894, 38663996, 38249932, 37523317, etc.). If this confounded our results, it would be predicted to overestimate the population requiring antibiotics, which we felt would be more reasonable than excluding this group, which might underestimate the patients needing antibiotic therapy. Especially as the harm would likely be perceived to be greater in overlooking these patients than falsely including them, we chose to include this code as well. Even so, few patients met these criteria, emphasizing how rare progression to systemic infection is, which just reiterates how reasonable it might be to implement decision support tools such as might be facilitated by machine learning, to reduce rampant antibiotic overprescription.

2. *Although the study assessed the impact of 16 features on the model, it did not select from the 16 features included in the model. If a subset of optimal features could be screened out from the 16 features, that is, by removing some redundant features, the model's performance might be improved.*

**Response:** We thank the reviewer for this comment. We did a detailed exploration into feature correlations (Figure S3), and found the feature ‘UA_MICRO’ to be redundant. The correlation of the remaining features was less than 0.5. We have added a new section on feature selection analysis to describe this process.

***Reviewer #2:*** *Please see the attached reviewer report for the full comments.*

*Major comments:*

*1. Abstract: Our models demonstrated high predictive performance with an area under the ROC curve (AUC) of 79.5% AUC and an area under the precision-recall curve (APR) of 13% APR for reencounters" - PR AUC of 13% sounds not high and ROC is less relevant for imbalance binary classification as mentioned in the Method section. Can you explain the claim that these results are high predictive performance? In addition, please clarify what are the baselines that the results with high predictive performance are compared to.*

**Response:** We have revised the abstract to be more precise: "Our models achieved significant improvement over baseline performance (APR = 0.004), with random forests reaching an APR of 0.15 for reencounters and 0.31 for urosepsis prediction. While these APR values may appear modest, they represent substantial predictive power given the extreme rarity of positive cases (0.4%) in our dataset." We have also added baseline comparisons in our Methods and Results sections, including a majority class classifier that demonstrates the difficulty of the prediction task. Our new Supplementary Figure 4 illustrates how our models substantially outperform these baselines.

*2. Introduction: There is a previous work by Zhang et al. (2021) that appears highly relevant to this research but is not mentioned in the related work section of the introduction.*

**Response:** We thank the review for this catch, we have modified the introduction to include this.

*3. Please justify the choice of these three machine learning models: logistic regression, decision tree, and random forest. For example, explain why XGBoost was not used.*

**Response:** We have modified the methods section to include justifications of the choice of ML models. We used logistic regression as a baseline, decision tree as a more complex tree-based model that can handle non-linear dependencies, and finally random forests as an ensemble model. We also implemented the XGBoost model, however since the results were very similar to random forests, we did not report it for simplicity.

*4. Have the authors considered any approaches to address the data imbalance? in a scenario of significant data imbalance, exploring approaches that address imbalanced datasets is highly relevant and essential to include in the manuscript.*

**Response:** We thank the reviewer for this important point about class imbalance. We have thoroughly investigated this issue by implementing and comparing multiple sampling strategies including SMOTE, random oversampling, and random undersampling. We have added a new section in Methods describing our approach to handling class imbalance, and included a new supplementary figure (Supplementary Figure 4) comparing model performance across these different strategies. Interestingly, random oversampling achieved the best performance (APR = 0.20) compared to SMOTE (APR = 0.11) and undersampling (APR = 0.03).

*5. Missing baselines: consider adding a random classifier and an always positive or negative classifier as baselines for comparison.*

**Response**: As mentioned above, we have added baseline comparisons in our Methods and Results sections, including a majority class classifier that demonstrates the difficulty of the prediction task. Our new Supplementary Figure 4 illustrates how our models substantially outperform these baselines.

*6. The reproducibility of this work is limited since the dataset is private and the code is not available currently. Therefore, consider applying your method to other datasets as well, especially public datasets under basic restrictions such as MIMIC-IV or eICU-CRD if possible. Additionally, reporting the results of another dataset can enhance the generalizability of this work.*

**Response:** Thank you for this recommendation. We are currently planning additional analyses refining and assessing the utility of this model as a clinical decision support tool. While the available databases you mention would be interesting to explore, the major purpose of this examination was to evaluate patients seen in an outpatient, non-acute setting to inform decisions for clinicians in these environments. There exist numerous other studies using machine learning to predict sepsis risk in a population presenting to the emergent care setting, but this population is fundamentally different than those presenting non-acutely to primary care and community health center environments. While the existing literature well documents the significantly increased risk of a sepsis diagnosis in patients who present for emergency care, there is little information on non-emergent, outpatient settings. The strength of our dataset is our ability to examine patients presenting to more than 200 outpatient medical practices and then trace their admissions and representations over time. It would be unlikely for these other available databases to provide the same results as we already know these are not equivalent populations. This report seeks to inform on the management of these less acute patients, providing insight into these differences and potential guidance to manage this fundamentally different population.

*7. Discussion, paragraph two: "but demonstrated that random forests were the best performing models, both in terms of ROC and model interpretability" - In a task where the data is highly imbalanced, ROC indeed is not relevant. Moreover, I think ROC performance can be misleading for that task given the extreme data imbalance. In light of this, I think it is better to remove this metric from the results. APR is more relevant, and reporting the precision and recall of the positive label (at a specific threshold) might also be interesting for real-world scenarios.*

**Response:** We thank the reviewer for this comment. We would like to respectfully disagree. There is recent work that reviews over 1.5 million scientific papers to refute this claim on AUPRC being a superior metric as compared to AUROC for class imbalance. (<https://arxiv.org/abs/2401.06091>)

***Reviewer #3:*** *The manuscript reports of a novel, systematic study of a problem that was not well address before. ML-based prediction of urosepsis is important can could have a significant impact on patients' lives. We believe this is a very promising study and we encourage the authors to further improve it following the comments below.*

1. *The authors correctly suggest that their dataset is significantly imbalanced (0.4% of the class of interest). AS we see it, the authors do not do enough in their study in response to this imbalance. One response it the use of precision-recall curve. This response is good to have a better metric to measure the quality of the results, and we are happy for the authors to have used it. Another response by the authors is data resampling, however resampling has a*

*limited effect in the case of 0.4% vs. 99.6%. There should likely be both oversampling of the minority class and under-sampling of the majority class. Also, some synthetic data can be added to the minority class. In addition, the small number of minority class records might lead to overfitting. The authors did not check this. Or maybe there is a possibility to get more real data? To summarize the imbalance problem - the authors need to carefully refer to all of the risks associated with this imbalance and suggest what they do to address each, as needed.*

**Response:** We thank the reviewer for this important point about class imbalance. We have thoroughly investigated this issue by implementing and comparing multiple sampling strategies including SMOTE, random oversampling, and random undersampling. We have added a new section in Methods describing our approach to handling class imbalance, and included a new supplementary figure (Supplementary Figure 4) comparing model performance across these different strategies. Interestingly, random oversampling achieved the best performance (APR = 0.20) compared to SMOTE (APR = 0.11) and undersampling (APR = 0.03).

2. *In the methods section, the authors suggest that they use logistic regression, decision trees, and random forests, for their ML analysis. But once we move to the results section, we find out that neural networks were used too. This is inconsistent. Also, if ANNs were used, more details about them are needed: which type of ANN was used? Why that type? More details on the other models could be useful too, to facilitate study repeatability.*

**Response:** We thank the reviewer for this comment. We have updated the machine learning analysis and described the ANN as well as other models in detail.

Additional Comments from Reviewer #2:

1 Abstract

*1. The abstract is too lengthy and should be summarized to include only the main points. Please ensure that the manuscript meets with the submission guidelines.*

**Response**: We have shortened the abstract to follow the submission guidelines.

*2. "Our models demonstrated high predictive performance with an area under the ROC curve (AUC) of 79.5% AUC and an area under the precision-recall curve (APR) of 13% APR for reencounters" - PR AUC of 13% sounds not high and ROC is less relevant for imbalance binary classification as mentioned in the Method section. Can you explain the claim that these results are high predictive performance? In addition, please clarify what are the baselines that the results with high predictive performance are compared to.*

**Response:** We have revised the abstract to be more precise: "Our models achieved significant improvement over baseline performance (APR = 0.004), with random forests reaching an APR of 0.15 for reencounters and 0.31 for urosepsis prediction. While these APR values may appear modest, they represent substantial predictive power given the extreme rarity of positive cases (0.4%) in our dataset." We have also added baseline comparisons in our Methods and Results sections, including a majority class classifier that demonstrates the difficulty of the prediction task. Our new Supplementary Figure 4 illustrates how our models substantially outperform these baselines.

*3. Consider to add to the Abstract the names of the Machine Learning models that were used for the predictions.*

**Response:** We have added the names of the ML models to the abstract.

*2 Introduction*

*1. There is a previous work by Zhang et al. (2021) [1] that appears highly relevant to this research but is not mentioned in the related work section of the introduction.*

**Response:** We thank the review for this catch, we have modified the introduction to include this.

*3 Methods*

*1. Please improve the resolution of the figures, such as Figure 1 from this section, as well as Figures 2, 3, and 4.*

**Response:** We thank the reviewer for this comment, we have attached high resolution with the revision

*2. Methods, third paragraph: "We split our examination into two major analyses. First, we predicted the risk of a patient with a UTI being admitted to the hospital for any reason in a 7-day window. Next, we narrowed down our predictor variable to consider only reencounters that were demonstrated cases of urosepsis" - Adding an ablation study with a baseline that predicts directly without the first step ("First, we predicted the risk of a patient with aUTI being admitted to the hospital for any reason"), can be beneficial for analysis and comparison.*

**Response:** We thank the reviewer for this insight. Please see below for further explanation. As for additional analyses, unfortunately an ablation study is out of the scope of this work.

*3. In addition to the previous comment, please provide a clarification on the hypothesis behind splitting this examination into two steps.*

**Response**: In previous examination of urosepsis risk in outpatient populations (e.g., Gharbi et al, BMJ, 2019, PMID: 30814048), many groups use all readmissions as a metric for failure of the outpatient management of UTI. We feel there is a problem with this definition, as many of the patients presenting to outpatient clinics with vague complaints attributed to UTI may be admitted to the hospital later because their initial presenting symptoms were not in fact due to urinary infections. Thus, this definition drastically overestimates of treatment failure, as many of the readmissions are as a result of the misdiagnosis of UTI, not failure to treat it. However, even though we fundamentally disagree with this definition of treatment failure, we wanted to include this analysis in our approach to derive a model using this metric because it has been commonly evaluated throughout the literature (which may be due to the absence of a diagnostic code indicating urosepsis). To facilitate better clinical decision-making, however, we felt the evaluation of reencounters that were documented urosepsis cases was more informative. As there is no diagnostic code for urosepsis, we attempted to reproduce the diagnostic criteria for this, which combined the documentation of sepsis/bacteremia/systemic signs of inflammation with a positive urine culture. This definition is imperfect as well, as many patients, particularly older patients, may have a positive urine culture even without a urinary infection, while others may not have had a urine culture obtained. Given the available data, however, we felt this was the most reliable way to predict the outcome. As this is also an imperfect definition, we presented both analyses.

*4. The train and test split is in a 75:25 ratio, and the validation set was later used for hyperparameter tuning. Please clarify the source of the validation set.*

**Response**: The dataset was randomly shuffled and split into training (75%) and test (25%) sets. Within the training set, k-fold cross-validation was used for hyperparameter tuning, while the test set was held out for final model evaluation.

*5. Consider adding general statistics that provide an overview of the task and dataset. For example, include the percentage of UTI patients diagnosed with urosepsis and those who are not. This will give a better estimate of how imbalanced the classification task is. The analysis of the Results (Data and data processing subsection) is more relevant to the Method section.*

**Response**: We thank the reviewer for this comment, we have listed these characteristics in table 1.

*6. Please justify the choice of these three machine learning models: logistic regression, decision tree, and random forest. For example, explain why XGBoost [2] was not used.*

**Response:** We have modified the methods section to include justifications of the choice of ML models. We used logistic regression as a baseline, decision tree as a more complex tree-based model that can handle non-linear dependencies, and finally random forests as an ensemble model. We also implemented the XGBoost model, however since the results were very similar to random forests, we did not report it for simplicity.

*7. The data is very imbalanced. Adding an approach to augment the dataset with more positive examples can enhance the contribution of this work.*

**Response:** We thank the reviewer for this important point about class imbalance. We have thoroughly investigated this issue by implementing and comparing multiple sampling strategies including SMOTE, random oversampling, and random undersampling. We have added a new section in Methods describing our approach to handling class imbalance, and included a new supplementary figure (Supplementary Figure 4) comparing model performance across these different strategies. Interestingly, random oversampling achieved the best performance (APR = 0.20) compared to SMOTE (APR = 0.11) and undersampling (APR = 0.03).

*8. Have the authors considered any approaches to address the data imbalance? in a scenario of significant data imbalance, exploring approaches that address imbalanced datasets is highly relevant and essential to include in the manuscript.*

**Response:** Please see the above answer.

4 Results

*1. There are some repetitions in the paper. For example, the train-test split ratio is mentioned both in the Methods and Results sections.*

**Response**: We have removed the split ratio from the Results for readability.

*2. Adding a caption to Figure 2 can enhance readability.*

**Response**: We have added a caption to figure 2.

*3. Missing baselines: consider adding a random classifier and an always positive or negative classifier as baselines for comparison.*

**Response**: We have added the performance of a random classifier as a baseline.

*4. The reproducibility of this work is limited since the dataset is private. Therefore, consider applying your method to other datasets as well, especially public datasets under basic restrictions such as MIMIC-IV or eICU-CRD if possible. Additionally, reporting the results of another dataset can enhance the generalizability of this work.*

**Response:** Thank you for this recommendation. We are currently planning additional analyses refining and assessing the utility of this model as a clinical decision support tool. While the available databases you mention would be interesting to explore, the major purpose of this examination was to evaluate patients seen in an outpatient, non-acute setting to inform decisions for clinicians in these environments. There exist numerous other studies using machine learning to predict sepsis risk in a population presenting to the emergent care setting, but this population is fundamentally different than those presenting non-acutely to primary care and community health center environments. While the existing literature well documents the significantly increased risk of a sepsis diagnosis in patients who present for emergency care, there is little information on non-emergent, outpatient settings. The strength of our dataset is our ability to examine patients presenting to more than 200 outpatient medical practices and then trace their admissions and representations over time. It would be unlikely for these other available databases to provide the same results as we already know these are not equivalent populations. This report seeks to inform on the management of these less acute patients, providing insight into these differences and potential guidance to manage this fundamentally different population.

*5. Consider to include the confidence intervals as part of the results.*

**Response**: We thank the reviewer for this comment, we have modified the manuscript to include confidence intervals.

5 Discussion

*1. Paragraph two: "but demonstrated that random forests were the best performing models, both in terms of ROC and model interpretability" - In a task where the data is highly imbalanced, ROC indeed is not relevant. Moreover, I think ROC performance can be misleading for that task given the extreme data imbalance. In light of this, I think it is better to remove this metric from the results. APR is more relevant, and reporting the precision and recall of the positive label (at a specific threshold) might also be interesting for real-world scenarios.*

**Response:** We thank the reviewer for this comment. We would like to respectfully disagree. There is recent work that reviews over 1.5 million scientific papers to refute this claim on AUPRC being a superior metric as compared to AUROC for class imbalance. (<https://arxiv.org/abs/2401.06091>)

*2. It is worth adding to the discussion whether, in your opinion, a practical application of predicting this task using this model is possible, considering the results, and how it compares to the current situation.*

**Response:** We thank the reviewer for this comment. We have modified the discussion to address this.

*3. Error analysis can be beneficial to understand the errors of the models. For example, it can be interesting to see what errors the Random Forest made in Figure 2B.*

**Response**: We thank the reviewer for this comment, this is out of scope of this work

*4. A more comprehensive discussion is needed to thoroughly analyze the result graphs. For example, how do you explain the very low accuracy in logistic regression, and what is the practical effect of the metrics, particularly in Figure 2B? Can a Random Forest model that achieves low recall and high precision be beneficial?*

**Response**: We thank the reviewer for this comment. We have modified the discussion to address this.

*5. In addition, the analysis lacks references to other baselines that were not tested in this paper, such as a model that always gives the same answer or a random classifier.*

**Response**: We have added a baseline model (Figure S4)

*6. It is worth comparing the results achieved by humans in this task with those presented in the manuscript.*

**Response**: We thank the reviewer for this comment, this is out of scope of this work
