# Supplementary material for: Machine learning for the prediction of urosepsis using electronic health record data
Source: PLOS Digit Health. 2025 Jul 3;4(7):e0000896. doi: 10.1371/journal.pdig.0000896 (PMC12225808; doi:10.1371/journal.pdig.0000896)
Supplement: Attachment — Submitted filename: Responses to Reviewers.docx [file pdig.0000896.s010.docx]

Responses to Reviewers

PLOS Digital Health: Machine learning for the prediction of urosepsis using electronic health record data

Reviewer #3: I am happy with the authors' responses to my comments - they fully address them. I have comments regarding Figure S4: 1. the fonts used are too small; 2) the legend in the left sub-figure covers hides parts of the curves, and therefore should be repositioned.

*We agree this was not optimal. We have reworked the figure to address your concerns and thank you for improving the clarity of the figure and paper.*
